# Antibody–Drug Conjugates in Breast Cancer: Ascent to Destiny and Beyond—A 2023 Review

**DOI:** 10.3390/curroncol30070474

**Published:** 2023-07-06

**Authors:** Tian Xiao, Sanji Ali, Danilo Giffoni M. M. Mata, Ana Elisa Lohmann, Phillip S. Blanchette

**Affiliations:** 1 Schulich School of Medicine, Western University, London, ON N6A 5C1, Canada; 2 Division of Medical Oncology, Department of Oncology, London Health Sciences Centre, Western University, London, ON N6A 5W9, Canada

**Keywords:** antibody–drug conjugate (ADCs), breast cancer, HER2-low

## Abstract

Antibody–drug conjugates (ADCs) are revolutionizing cancer treatment, adding another important new class of systemic therapy. ADCs are a specially designed class of therapeutics that target cells expressing specific cancer antigens using directed antibody–drug delivery and release a cytotoxic chemotherapeutic payload. Over the past two decades, improvements in ADC design, development, and research, particularly in breast cancer, have led to several recent landmark publications. These advances have significantly changed various treatment paradigms and revamped traditional classifications of breast cancer with the introduction of a potential new subtype: “HER2-low”. This review will focus on several ADCs developed for breast cancer treatment, including trastuzumab emtansine (T-DM1), trastuzumab deruxtecan (T-DXd), sacituzumab govitecan (SG) and other newer emerging agents. It will provide an overview of the role of ADCs in breast cancer and discuss the opportunities and challenges they present. Additionally, our review will discuss future research directions to improve the selection of targets, combination therapies, and aim to improve drug safety. Important first-line metastatic and adjuvant clinical trials are underway, which may expand the role of ADC therapy in breast cancer. We foresee ADCs driving a new era of breast cancer treatment, adding to the steady incremental survival advantage observed in recent years.

## 1. Introduction: Development and History of ADCs in Breast Cancer

Targeted drug delivery was originally described by Dr. Paul Ehrlich, known as the ‘magic bullet concept’, whereby therapeutic agents could be directed precisely against microbes or cancerous cells nearly 100 years ago [1]. Nonetheless, for much of the 20th century, targeted cancer therapy was not feasible and cytotoxic chemotherapy remained the mainstay of treatment. The first breakthrough in the development of ADCs was monoclonal antibody therapy directed against cancer cells. Antibodies, also known as immunoglobulins, are proteins of the humoral immune system produced in response to a foreign antigen. In the context of therapeutic use against a specific cancer antigen, scientists can create a specific antibody clone called a monoclonal antibody. Through selective interaction with cancer cell antigens, monoclonal antibodies can directly target cancer cells with minimal interference with normal tissue function. The concept of monoclonal antibody-directed cancer therapy is exemplified by trastuzumab, a monoclonal antibody targeted against the human epidermal growth factor receptor 2 (HER2) receptor and a mainstay of anti-HER2-directed therapy for breast cancer [2]. However, trastuzumab is inadequate as a monotherapy and requires concurrent administration with chemotherapy for maximum therapeutic benefit. Additional advancements in technology, development, and research were required before targeted antibodies could be directly linked to transport cytotoxic chemotherapy drugs into cancer cells. Further progress culminated in the first regulatory approval for an ADC, gemtuzumab ozogamicin, which received accelerated approval for acute myeloid leukemia in 2000. However, the approval was later withdrawn due to failure to show clinical benefit in the post-marketing phase [3].

The ADC complex consists of a tumoral antigen-specific antibody connected via a linker to a potent cytotoxic agent known as the payload [4]. The cytotoxic effect of ADCs on cancer cells may act through the following mechanisms: (1) directed cell cytotoxicity through internalization of the ADC complex into the cell via recognition of specific cancer antigens by the antibody, followed by the cleavage of the stable linker and dissociation of the cytotoxic payload, (2) direct antibody-mediated effects either through inhibiting downstream signal transduction of the antigen receptor or antibody-dependent-mediated cellular cytotoxicity (ADCC), and (3) the bystander effect, whereby upon cell death, the cytotoxic payload is released into the surrounding tumor bed. 

The efficacy of ADCs is determined by numerous factors, including the affinity to the antigen target, effective release of the linker, linker stability to prevent premature release of payload leading to off-site toxicity, and potency and quantity of the payload [4]. The newer generations of ADCs have shown superior results in terms of disease control by optimizing many of these factors, including the introduction of the bystander effect.

**Figure 1:**
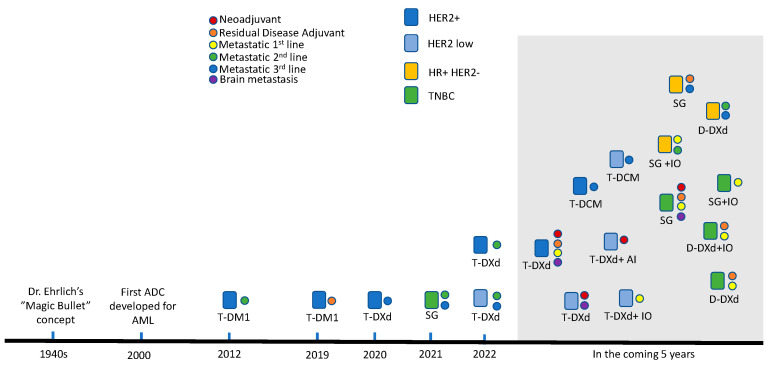
Timeline of current and future antibody–drug conjugate (ADC) development in breast cancer. AI: Aromatase inhibitor; AML: Acute myeloid leukemia; Dato-DXd: Datopotamab deruxtecan; HER2+: HER2 positive; HER2−: HER2 negative; HR+: Hormone receptor positive; IO: Immunotherapy; SG: Sacituzumab govitecan; T-DCM: Trastuzumab duocarmazine; T-DM1: Trastuzumab emtansine; T-DXd: Trastuzumab deruxtecan; TNBC: Triple negative breast cancer.

Trastuzumab emtansine (T-DM1) was the first ADC approved in breast cancer in 2013 [5]. More than half a decade later, two more ADCs have received regulatory approvals in the setting of breast cancer, including trastuzumab deruxtecan (T-DXd), and sacituzumab govitecan (SG). Both T-DXd and SG have demonstrated bystander effects and exceptional tumor control in breast cancer. The growth of the ADC landscape is exponential, with nearly 20 clinical trials now investigating these two ADCs alone as they move into earlier lines of treatment, not including others which we will discuss below (Figure 1). We present an overview of the specific molecular structures of selected ADCs (Table 1), as well as the summary of results from the ADC clinical trials currently shaping the landscape of treatment in breast cancer (Table 2) and future ongoing clinical trials of potentially important clinical relevance (Table 3). 

## 2. Current Landscape of ADCs in Breast Cancer

### 2.1. Trastuzumab Emtansine (T-DM1)

T-DM1 is an ADC where the antibody trastuzumab is joined with a microtubule inhibitor, emtansine, at a drug-to-antibody ratio (DAR) of 1:3.5 via a non-reducible thioether linker that is stable in both systemic circulation and the tumor microenvironment [6]. Due to emtansine’s positive charge and membrane impermeability after release into the cell, there is no bystander effect. It is currently approved in both the adjuvant and second-line metastatic setting for HER2-positive (HER2+) breast cancer.

T-DM1 was first approved as a second-line treatment for HER2+ breast cancer based on the results of the EMILIA trial, which demonstrated a significant improvement in progression-free survival (PFS) for patients treated with T-DM1 as compared to capecitabine and lapatinib (9.6 vs. 6.4 months, hazard ratio (HR) = 0.65, 95% confidence interval (CI): 0.55–0.77; *p* < 0.001) [7]. The absolute overall survival (OS) benefit of T-DM1 was approximately 4 months with a 25% risk reduction in death when compared to the control arm (29.9 vs. 25.9 months, hazard ratio (HR) = 0.75, 95% confidence interval (CI): 0.64–0.88) [8]. However, in the MARIANNE trial, T-DM1 failed to show a survival advantage of T-DM1 and pertuzumab in comparison to trastuzumab plus taxane chemotherapy in the first-line setting for metastatic HER2+ breast cancer [9]. 

In the adjuvant KATHERINE trial, early-stage HER2+ breast cancer patients who had residual tumor post-neoadjuvant treatment with trastuzumab and taxane chemotherapy were randomized to receive either 14 cycles of adjuvant T-DM1 or trastuzumab [10]. The results from the KATHERINE trial demonstrated a significant improvement in invasive disease-free survival (IDFS) among patients treated with T-DM1 versus trastuzumab (IDFS at 3 years: 88.3% vs. 77%, HR = 0.50, 95% CI: 0.39–0.64, *p* < 0.001). This was the first positive ADC trial in the adjuvant setting in breast cancer and established T-DM1 as the preferred adjuvant therapy for HER2+ breast cancer patients with residual disease after completion of neoadjuvant chemotherapy [11].

### 2.2. Trastuzumab Deruxtecan (T-DXd)

Deruxtecan is a drug component derivative of exatecan mesylate, a topoisomerase I inhibitor with activity close to ten times that of 7-ethyl-10-hydroxycamptothecin (SN38), the active metabolite of irinotecan. T-DXd’s payload is linked via a tetrapeptide-based cleavable linker, characterizing this ADC to be highly stable in plasma [12]. Deruxtecan is membrane permeable, which allows its diffusion out of the target cell to exert its cytotoxic effects on nearby cancer cells via the bystander effect [13]. T-DXd is approved in both second-line treatment and for patients who have received prior treatment with T-DM1 for metastatic HER2+ breast cancer. T-DXd is also approved for treatment in a new category of advanced breast cancer where HER2 expression is low (HER2-low) defined by either HER2 1+ or 2+ testing by immunohistochemistry (IHC) and a negative in-situ hybridization (ISH) testing.

In the phase III DESTINY-Breast02 trial, T-DXd demonstrated a significant PFS benefit in the third-line setting after disease progression with T-DM1 when compared to the combination of capecitabine and lapatinib or capecitabine and trastuzumab in HER2+ breast cancer [14]. Subsequently, the DESTINY-Breast03 trial compared T-DXd to T-DM1 as second-line treatments for advanced HER2+ breast cancer following treatment with first-line trastuzumab, pertuzumab and taxane chemotherapy (62% of participants were previously treated with pertuzumab) [15]. In the updated survival results of DESTINY-Breast03, there was a significant improvement in PFS in patients treated with T-DXd as compared to T-DM1 (28.8 vs. 6.8 months, HR = 0.33, 95% CI: 0.26–0.43; *p* < 0.001) [16]. The radiographic response rates for T-DXd versus T-DM1 were as follows: (1) the objective response rate (ORR) was 79% versus 35% and (2) the complete response rates (CRR) were 21% versus 10%, respectively. Median OS has not been reached for either drug at the 4-year interim analysis. Although the drug-related interstitial lung disease (ILD) rates were higher in T-DXd (15.2%) versus T-DM1 (3.1%), the majority of the ILD reactions were low grade. In terms of CNS disease, the ORR and CRR were higher for T-DXd as compared to T-DM1 (ORR: 67.4% vs. 20.5% and CRR: 27.8% vs. 2.8%), respectively [17]. Given these results, T-DXd is now the preferred second-line treatment option for metastatic HER2+ breast cancer.

The traditional classification of HER2-negative breast cancer is defined by negative HER2 expression by IHC and gene amplification by ISH (or commonly known as FISH when using fluorescent probes). HER2-negative breast cancer was identified by either the absence of HER2 protein staining (IHC 0); incomplete HER2 staining (IHC 1+); or moderate staining (IHC 2+) and negative testing for HER2 amplification by ISH. Given the new bystander effect exhibited by T-DXd, it was decided that the HER2 biomarker was able to be leveraged as a vehicle for the payload, even when there is a lower degree of HER2 overexpression. DESTINY-Breast04, published in July 2022, has confirmed this concept of targeting “HER2-low” positivity with this new ADC [18]. Five-hundred and fifty-seven patients who were classified as HER2-low (58% (IHC 1+), 42% (IHC 2+ and FISH negative)) and previously treated with anti-estrogen therapy (if hormone receptor-positive (HR+)) and one to two lines of chemotherapy were randomized to receive either T-DXd or the physician’s choice of chemotherapy (capecitabine, eribulin, gemcitabine, paclitaxel, or nab-paclitaxel) [18]. In a subset of patients with HR+ HER2-low breast cancer (88% of study participants), patients treated with T-DXd as compared to the physician’s choice of treatment had a significant improvement in PFS (10.1 vs. 5.4 months, HR = 0.51, 95% CI: 0.4–0.64; *p* < 0.001) and OS (23.9 vs. 17.5 months, HR = 0.64, 95% CI: 0.48–0.86; *p* = 0.003). In the entire cohort irrespective of hormone receptor status, T-DXd continued to demonstrate superiority as compared to the physician’s choice of treatment (PFS: 9.9 vs. 5.1 months, HR = 0.50, 95% CI: 0.40–0.63; *p* < 0.001 and OS: 23.4 vs. 16.8 months, HR = 0.64, 95% CI: 0.49–0.84; *p* = 0.001, respectively) [18]. Based on the DESTINY-Breast04 trial, T-DXd was recently approved for patients with unresectable/metastatic HER2-low (IHC 1+ or IHC 2+ and HER2 ISH-negative) breast cancer who have either received one line of chemotherapy in the metastatic setting or progressed within 6 months of adjuvant chemotherapy [19].

New clinical trials for HER2+ breast cancer populations may also add additional indications for T-DXd as studies are ongoing in the neoadjuvant setting (DESTINY-Breast11) [20], adjuvant setting for patients with residual disease compared to T-DM1 (DESTINY-Breast05) [21], and as a first-line therapy for metastatic disease comparing T-DXd versus pertuzumab, trastuzumab and taxane chemotherapy (DESTINY-Breast09) [22]. T-DXd is also undergoing a phase III study on patients with HER2+ breast cancer and central nervous system (CNS) metastasis (DESTINY-Breast12 [23]) following favorable intracranial activity (ORR_intracranial_ 46.2%) observed in the DEBBRAH trial, a single arm phase II study on patients with HER2-positive breast cancer and active CNS metastasis receiving T-DXd [24]. Additionally, the DESTINY-Breast06 clinical trial is further investigating T-DXd among patients with HR+ and HER2-low disease in breast cancer patients who have progressed on prior endocrine therapy without previous chemotherapy, compared with the physician’s choice of chemotherapy (capecitabine, paclitaxel or nab-paclitaxel) [25]. This study has also included an ultra-low HER2 classification (HER2 IHC >0 and IHC<1+), a group that may also benefit as demonstrated in the DAISY trial, a phase II study evaluating T-DXd across all levels of HER2 expression in patients with metastatic/unresectable breast cancer. In DAISY, T-DXd demonstrated a best overall response rate of 30% (95% CI: 16–47%) and a median PFS of 4.2 months (95% CI: 2–5.7) even in patients with metastatic HER2-negative (IHC 0) disease [26].

### 2.3. Sacituzumab Govitecan (SG)

SG is designed as a humanized monoclonal antibody against trophoblastic cell-surface antigen-2 (TROP-2), a receptor discovered in trophoblasts 40 years ago [27]. Sacituzumab is also linked to the topoisomerase I inhibitor component SN-38 via a hydrolysable linker [28]. Like T-DXd, SG also has a high DAR of 7.6:1, and like T-DXd, it also exerts a bystander effect. It is approved in the third-line setting for metastatic triple negative breast cancer (mTNBC) and in later line therapy for metastatic HR+ HER2− breast cancer. 

Triple negative breast cancer (TNBC) is defined by estrogen and progesterone receptor staining less than 1% of cancer cells and HER2 testing that is negative for protein expression or gene amplification (IHC 0 or 1+, or IHC 2+ and ISH negative) [29]. Systemic treatment options for mTNBC have remained poor with chemotherapy, offering a modest clinical benefit and immune checkpoint inhibitor therapy only providing a small incremental survival advantage among selected patients who are programmed death ligand-1 (PD-L1)-positive (combined positive score (CPS) ≥ 10) [30]. SG was evaluated in the ASCENT trial in the third-line setting in mTNBC after disease progression on at least two lines of chemotherapy, one of which included taxane [31]. In the ASCENT trial, SG was compared to the investigator’s choice of eribulin, capecitabine, gemcitabine, or vinorelbine and a significant improvement in PFS (5.6 vs. 1.7 months, HR = 0.41, 95% CI: 0.32–0.52; *p* < 0.001) and OS (12.1 vs. 6.7 month, HR = 0.48; 95% CI: 0.38–0.59; *p* < 0.001) was observed. The ORR was 35% with SG as compared to 5% with chemotherapy. Currently, SG has demonstrated the largest significant clinical benefit observed in clinical trials for patients with heavily pre-treated mTNBC [32].

The ASCENT-03 and ASCENT-04 clinical trials are currently enrolling patients with untreated, locally advanced inoperable or first-line mTNBC. ASCENT-03 is investigating SG versus physician’s choice of therapy for patients whose breast tumors are PD-L1-negative (CPS < 10) [33]. Conversely, the ASCENT-04 trial combines SG and pembrolizumab versus the physician’s choice of therapy and pembrolizumab for patients whose breast cancers are PD-L1-positive (CPS ≥ 10) [34]. 

In HR+ HER2− breast cancer, once patients become endocrine therapy refractory or when treatment options are limited, chemotherapy remains the mainstay of treatment aside from clinical trials. Although recent data from Destiny-Breast04 have shown promising results with T-DXd in HER2-low expressing breast cancers, treatment for patients with HER2-negative (IHC 0) tumors remains a challenge [35,36]. The latter group comprises of approximately 40% of the HER2–negative breast cancers [35,36]. The most recent TROPiCS-02 trial, still in abstract form, compared SG to the physician’s choice of chemotherapy (eribulin, capecitabine, gemcitabine, or vinorelbine) in patients with metastatic/unresectable HR+ HER2− breast cancer who have received at least one line of treatment, including endocrine therapy, a cyclin-dependent kinase 4/6 (CDK4/6) inhibitor, and taxane [37]. The second interim analysis demonstrated significant improvement favoring SG with regards to PFS (5.5 months vs. 4.0 months, HR = 0.66, 95% CI: 0.53–0.83; *p* = 0.0003) and OS (14.4 vs. 11.2 months, HR = 0.79, 95% CI: 0.65–0.96; *p* = 0.020). At the San Antonio Breast cancer Symposium (SABCS) 2022, the latest exploratory analysis showed a persistent effect across a wide range of Trop-2 expression levels [38].

SG is also being investigated in the adjuvant SASCIA clinical trial among patients with HER2-negative breast cancers with residual disease after neoadjuvant chemotherapy as compared to the physician’s choice (either capecitabine, platinum-based chemotherapy or observation) [39]. The usage of adjuvant CDK4/6 inhibitor, ET or pembrolizumab was allowed according to local guidelines. 

## 3. Emerging ADC Development

We have chosen to highlight the following two new ADCs: datopotamab deruxtecan and trastuzumab duocarmazine, which may be the next to emerge into routine clinical practice. There is a plethora of ADCs currently under development for breast cancer in phase 1/2 trials, which are beyond the scope of this clinically focused review. A number of emerging ADCs targeting novel tumor antigens are in development, including B7-H3 [40], B7-H4 [41] CD166 [42], HER3 [43], LIV1 [44], Nectin-4 [45], ROR1 [46], and ROR2 [47].

### 3.1. Datopotamab Deruxtecan (Dato-DXd)

Dato-DXd also targets TROP-2 and is combined with deruxtecan, a topoisomerase I inhibitor, via a tetrapeptide-based cleavable linker with a DAR of up to 4. Dato-DXd has also been shown to exert a bystander effect.

In TROPION-PanTumour01, a phase 1 dose-escalation study involving multiple solid tumor malignancies, Dato-DXd was studied in patients with TNBC and HR+ HER2– breast cancer patients who developed disease relapse from standard treatment or for whom no standard treatment was available. Patients had previously been exposed to three lines of therapy on average in the advanced disease setting and 30% of patients had prior exposure to an ADC with a topoisomerase I payload. In participants with TNBC (N = 44), the ORR was 32% and the median OS was 13.5 months (10.1–16.3 months), whereas in those who were ADC naïve (topoisomerase I payload), the ORR was 44% with OS of 14.3 months. The most common treatment-related adverse events were predominantly nausea and stomatitis events of low grade and no cases of ILD were reported [48].

Based on the promising early phase clinical trial results, Dato-DXd is entering a phase 3 TROPION-Breast01 clinical trial versus the investigator’s choice of chemotherapy (eribulin, capecitabine, vinorelbine, gemcitabine) in patients with inoperable/metastatic HR+ HER2− breast cancers who progressed on one or two lines of chemotherapy [49].

In mTNBC, TROPION-Breast02 is a phase 3 trial comparing Dato-DXd against physician’s choice of chemotherapy (eribulin, capecitabine, paclitaxel, nab-paclitaxel, carboplatin) in patients with inoperable/mTNBC who are not candidates for PD-1/PD-L1 therapy [50]. In those who are candidates for PD-1/PD-L1 therapy, the phase 1b/2 trial BEGONIA (N = 61) appraising Dato-DXd + durvalumab showed an ORR of 73.6% (95% CI: 59.7–84.7) in patients with unresectable/mTNBC in the first-line setting. Treatment responses were observed regardless of the degree of PD-L1 expression. The most common adverse events were nausea, stomatitis, alopecia, and fatigue. In this trial, 3.3% of patients developed ILD, which was limited to grade 1 only [51]. Based on the BEGONIA trial results, the TROPION-Breast05 will be the phase 3 trial evaluating Dato-DXd with durvalumab against chemotherapy plus pembrolizumab in first-line treatment for patients with mTNBC and who are PDL-1 positive (with tumor area positivity—TAP ≥ 10%).

In addition, TROPION-Breast03 is an upcoming phase 3 study, which compares Dato-DXd plus/minus durvalumab versus capecitabine and/or pembrolizumab in the residual disease setting for patients with early-stage TNBC without a pathologic complete response after neoadjuvant chemotherapy [52].

### 3.2. Trastuzumab Duocarmazine

Trastuzumab duocarmazine is a newer ADC formulated from trastuzumab linked to duocarmazine, a potent DNA minor-groove-binding alkylating agent by a cleavable linker, which also exhibits a bystander effect [53]. In comparison to T-DM1, it exhibits 10- to 70-fold anti-tumor activity in xenograft models, despite a lower DAR of 2.8 [53].

A phase 3 trial, called TULIP, randomized trastuzumab duocarmazine versus the physician’s choice (four choices in total: lapatinib and capecitabine, trastuzumab combined with either vinorelbine, capecitabine, or eribulin) in patients with metastatic HER2+ breast cancer who have progressed on two lines of HER2-directed therapy. The preliminary results report a superior PFS for trastuzumab duocarmazine as compared to the physician’s choice (7 vs. 4.9 months, HR = 0.64, 95% CI: 0.49–0.84; *p* = 0.002) [54].

For patients with metastatic HER2-low breast cancer, a phase 1 study in a dose-escalation/expansion trial is ongoing. The preliminary results show an ORR of 40% of patients with HER2-low, ER− breast cancer, ORR of 33% among patients with HER2+ breast cancer, and ORR of 28% among patients with HER2-low, HR+ breast cancer [55].

## 4. Discussion

### 4.1. Future Directions for Current ADC Therapy

ADC therapy is associated with improvement in outcomes in breast cancer across all receptor subtypes and may lead to the replacement of traditional cytotoxic chemotherapy treatment in the near future. However, many questions and challenges remain surrounding the optimization of ADC therapy in the future. In HER2+ and TNBC, the development of T-DM1, T-DXd, and SG has followed the traditional progression of clinical trial research. Studies such as EMILIA, DESTINY-02 and ASCENT trials among more heavily pre-treated metastatic patients showed significant clinical benefit, meriting further clinical trials in earlier disease settings [7,14,31]. In the coming years, we will also likely have a better understanding of T-DXd and SG’s role in first-line and adjuvant therapy. The potential role of ADCs in HR+ and HER2-low or HER2-negative breast cancer seems more complex and may be restricted to patients encountering resistance to previous endocrine therapy.

Among patients with mTNBC, the ASCENT-03 and ASCENT-04 clinical trials will determine SG’s role in first-line therapy and investigate the potential synergy with immune checkpoint inhibitor therapy [33,34]. The effectiveness of ADCs as adjuvant therapy for TNBC is less certain. Early results from the neoadjuvant NEOSTAR trial showed only a modest pCR rate of 30% compared to the neoadjuvant KEYNOTE-522 trial (chemotherapy and pembrolizumab), where pCR rates reached historical highs of 64.8% [56,57]. SG is also currently being studied in neoadjuvant TNBC patients concurrently with pembrolizumab [58] but it may be better suited to patients with residual disease post-neoadjuvant chemotherapy, which is currently being tested in the adjuvant SASCIA trial [39].

T-DXd is now the superior second-line treatment of HER2+ breast cancer, demonstrating an impressive survival benefit as compared to T-DM1 [16]. There are several factors that contribute to this benefit, such as a higher drug–antibody ratio and potentially improved payload efficacy. However, the most significant factor is likely the bystander effect. Despite these results, T-DXd will have a significant challenge to demonstrate superiority as compared to first-line pertuzumab, trastuzumab and taxane chemotherapy regimens. T-DM1 was unable to demonstrate superiority in the MARIANNE clinical trial and the updated results from the CLEOPATRA study demonstrating a median PFS of 18.7 months, OS of 57.1 months and a survival rate of 37% at 8 years are formidable [9,59].T-DXd will likely also be a potential promising adjuvant therapy. However, heterogeneous disease or mixed histology subtypes may represent a challenge in using ADCs as compared to conventional chemotherapy [60]. Many clinical trials in early and metastatic settings are underway but results will not be expected for several years. T-DXd also appears to be active in patients with CNS metastases and a phase II clinical trial combining T-DXd and tucatinib will be closely watched in the HER2Climb04 trial [61]. 

### 4.2. Toxicities of ADCs

The side effects of ADCs may reflect either the tumoral antigen-specific antibody or its cytotoxic payload. Trials to date have shown similar rates of reversible cardiomyopathy risk at less than 2–3% with trastuzumab-based ADCs in comparison to trastuzumab therapy [9,10,16], and no specific risk side effects have been identified with TROP-2 antagonism from sacituzumab. Ideally, the systemic side effects secondary to the cytotoxic payload are minimized as the antibody is required to be internalized into the cell and the linker molecule must be cleaved or degraded before the cytotoxic payload is released. The use of ADCs such as SG and T-DXd has been shown to be tolerable with manageable side effects, as the incidence of severe febrile neutropenia, anemia, nausea and diarrhea are all within 10% [15,31]. Nonetheless, 25% of patients required dose reductions in both the ASCENT and DESTINY-Breast-03 trials, despite the high utilization of G-CSF and early discontinuation rates, which ranged from 5 to 15% [16,31]. In addition, the hematologic toxicity of SG can also be exacerbated in patients with polymorphisms in uridine diphosphate glucuronosyltransferase 1A1 (Ex. homozygosity for UGT1A1*28) and in patients on medication causing inhibition of UGT1A1. T-DXd is also associated with a 15% risk of drug-induced ILD, warranting close monitoring and early intervention [16,18]. While the rates of serious ≥grade 3 pneumonitis are low, this adverse side effect is of concern, particularly if T-DXd was combined with immune checkpoint inhibitor therapy, radiation or explored further in adjuvant therapy [21,62]. There is a need for further studies investigating the safety, tolerability, and optimal drug dosing of ADC therapy among elderly patients and those with multiple medical co-morbidities. There also needs to be long-term safety data to evaluate the potentially irreversible toxicity, such as the risk of secondary malignancies. At present, the quality-of-life scores appear favorable against cytotoxic chemotherapy among heavily pre-treated breast cancer patients. Nonetheless, quality of life studies in clinical trials evaluating ADCs as adjuvant therapy and as first-line metastatic therapy will be needed. We also have a limited understanding of the late effects of ADC therapy, which will require more utilization and follow-up to evaluate. 

### 4.3. Hormone Receptor (HR)-Positive and HER2-Low/HER2-Negative Breast Cancer

T-DXd and SG have demonstrated significant survival benefits in HR+ and HER2-low and HER2-negative breast cancers that are refractory to endocrine therapy options. This is also a major development in HR+ breast cancers, paving the way for a new ADC era potentially without the use of traditional cytotoxic chemotherapy. The T-DXd results were particularly impressive when using a new classification of HER2-low breast cancer. Further clinical trials testing ADCs against endocrine therapy are unlikely. However, clinical trials in high-risk ER+ HER2-low and HER2-negative breast cancer in the adjuvant setting as opposed to cytotoxic chemotherapy are of interest. 

Further refinement of HER2 testing to limit potential misclassification may also be warranted. The interpretation of HER2 scoring based on IHC staining may vary by observer, potentially impacting patient drug eligibility [63]. Tumor heterogeneity and the use of archival tissue may also introduce inaccuracy, as HER2 expression may alter over time depending on systemic therapy exposure [64,65]. Regardless, data are emerging regarding the potential T-DXd benefit among patients without HER2 amplification and trials in HER2 “ultra-low” (i.e., HER2 IHC 0 with incomplete and faint staining in ≤10% of tumor cells) are underway [25,26]. SG may also have an advantage in the HER2-negative setting by targeting TROP2, an intracellular calcium signal transduction transmembrane glycoprotein [31]. Nonetheless, TROP2 expression has not been associated with treatment efficacy and our understanding of factors and biomarkers to predict responses to ADC therapy for individual patients remains incomplete.

### 4.4. Resistance Mechanisms and Drug Design

Comparative and sequencing studies will also be needed given the numerous ADCs in development and their unique properties. These studies should also be carefully designed based on our understanding of ADC resistance. Potential mechanisms of ADC resistance include the following: (1) downregulation of the ADC antigen target, (2) altered ADC internalization and trafficking, (3) overexpression of drug efflux pumps, (4) reduced cytotoxic action of the payload, and (5) apoptosis dysregulation [66,67]. Currently, SG resistance has been observed to be associated with the downregulation of TROP2 expression and mutations impacting anti-topoisomerase I of the payload SN-38 [68]. In the DAISY trial, the decrease in HER2 expression and *SLX4* loss involved in DNA maintenance and repair were associated with acquired T-DXd resistance [69]. Concurrent administration of ADCs with a variety of immunotherapeutic drugs and other targeted therapies may create synergy and delay resistance. Numerous ongoing trials are evaluating the combination of ADCs, immunotherapy, CDK4/6 inhibitors, and anti-estrogen therapies. Trials in patients with germline BRCA mutations with concurrent use of poly (adenosine diphosphate–ribose) polymerase (PARP) inhibitors may also be attractive. Novel ADCs continue to be developed with newer antigens and payloads, as well as the use of site-specific conjugation of payloads to antibodies with the objective of improving selective tumor uptake, payload potency, therapeutic index and toxicity [70]. The newer generation ADCs in development include bispecific ADCs, pro-body drug conjugates (an antibody prodrug that requires proteolytic cleavage for activation in the tumor microenvironment), radionucleotide–drug conjugates, immunostimulatory antibody–drug conjugates, and small molecular drug conjugates [71]. 

## 5. Conclusions

The dawn of the ADC era in breast cancer has arrived. ADC therapy is impacting all subtypes of breast cancer and defining a potential subclassification, “HER2-low”. In addition, with the capability of targeting specific cancer antigens beyond HER2, this is a step towards personalization of therapy. This new class of therapy holds great promise and will likely expand into first-line and adjuvant treatment settings. Regardless, toxicities are not insignificant and an improved understanding of sequencing, combination therapy, biomarker development, and resistance is needed. The development of newer generation ADCs that are already underway may help to further improve their precision, potency, and safety with ever greater potential and shape the landscape of breast cancer treatment in the future.

## Figures and Tables

**Table 1:** Characteristics of selected breast cancer antibody drug conjugates (ADCs).

ADC	Target	Antibody	Linker	Payload	DAR
Trastuzumab emtansine (T-DM1)	HER2	Trastuzumab	Non-Cleavable	Emtansine (Microtubule Inhibitor)	3.5
Trastuzumab deruxtecan (T-DXd)			Cleavable	Deruxtecan (Topoisomerase I Inhibitor)	8
Trastuzumab duocarmazine			Cleavable	Duocarmazine (Alkylating Agent)	2.8
Datopotamab deruxtecan	Trop-2	Datopotamab	Cleavable	Deruxtecan (Topoisomerase I Inhibitor)	4
Sacituzumab govitecan		Sacituzumab	Cleavable	Govitecan (Topoisomerase I Inhibitor)	7.6

DAR: Drug antibody ratio; HER-2: Human epidermal growth factor receptor 2; Trop-2: Trophoblast cell-surface antigen 2.

**Table 2:** Summary of phase III clinical trials for antibody–drug conjugates (ADCs) with regulatory drug approval for breast cancer.

ADC	Study	Setting	Phase	Treatment	Recurrence Outcome (Hazard Ratio, 95% CI; *p* Value)	OS (Hazard Ratio, 95% CI; *p* Value)
**HER2-positive**						
T-DM1	KATHERINE (2019)	Adjuvant (residual disease post-neoadjuvant)	3	T-DM1 vs. trastuzumab	3-year IDFS: 88.3% vs. 77.02% (HR = 0.50, 95% CI: 0.39–0.64; *p* < 0.001)	Data not mature
	EMILIA (2012)	2nd line metastatic	3	T-DM1 vs. lapatinib and capecitabine	PFS: 9.6 vs. 6.4 months (HR = 0.65, 95% CI: 0.55–0.77; *p* < 0.001)	29.9 vs. 25.9 months HR = 0·75 (95% CI: 0.64–0.88)
T-DXd	Destiny-Breast03 (2022)	2nd line metastatic	3	T-DXd vs. T-DM1	PFS: 28.8 vs. 6.8 months (HR = 0.33, 95% CI: 0.26–0.43; *p* < 0.0001)	Data not mature
	Destiny-Breast02 (2023)	3rd line metastatic	3	T-DXd vs. physician’s choice of therapy	PFS: 17.8 vs. 6.9 months (HR = 0.36, 95% CI: 0.28–0.45; *p* < 0.0001)	39.2 vs. 26.5 months (HR = 0.66, 95% CI: 0.50–0.86; *p* = 0.0021)
**HER2-low, Hormone receptor-positive and negative**						
T-DXd	Destiny-Breast04 (2022)	2nd or 3rd line metastatic	3	T-DXd vs. physician’s choice of therapy	PFS: 9.9 vs. 5.1 months (HR = 0.50, 95% CI: 0.40–0.63; *p* < 0.001)	23.4 vs. 16.8 months (HR = 0.64, 95% CI: 0.49–0.84; *p* = 0.001)
**HER2-negative, Hormone receptor-positive**						
SG	TROPiCS-02 (2022) *	3rd or later line metastatic	3	SG vs. physician’s choice of therapy	PFS: 5.5 vs. 4.0 months (HR = 0.66, 95% CI: 0.53–0.83; *p* = 0.0003)	14.4 vs. 11.2 months (HR = 0.79, 95% CI: 0.65–0.96; *p* = 0.02)
**Triple Negative (TNBC)**						
SG	ASCENT (2022)	2nd or 3rd line metastatic	3	SG vs. physician’s choice of therapy	PFS: 5.6 vs. 1.7 months (HR = 0.41, 95% CI: 0.32–0.52; *p* < 0.001)	12.1 vs. 6.7 months (HR = 0.48, 95% CI: 0.38–0.59; *p* < 0.001)

All current published data are phase III RCTs. ADC: Antibody–drug conjugate; CI: Confidence interval; HER2-negative: Human epidermal growth factor receptor 2-negative; HER2-positive: Human epidermal growth factor receptor 2-positive. HR: Hazard ratio; IDFS: Invasive disease-free survival; OS: Overall survival; PFS: Progression-free survival; SG: Sacituzumab govitecan; T-DXd: Trastuzumab deruxtecan; T-DM1: Trastuzumab emtansine; TNBC: Triple negative breast cancer. * Presented as oral presentation and abstract. Awaiting publication.

**Table 3:** Selected ongoing clinical trials for antibody–drug conjugates (ADCs) in breast cancer.

ADC	Receptor Status	Study	Setting	Phase	Intervention	Primary End Point
**HER2-positive**						
T-DXd	DESTINY-Breast05		Adjuvant (residual disease post-neoadjuvant chemotherapy)	3	T-DXd vs. T-DM1	IDFS
	DESTINY-Breast09		1st line metastatic	3	T-DXd ± pertuzumab vs. pertuzumab, trastuzumab and taxane chemotherapy	PFS
	DESTINY-Breast11		Neoadjuvant	3	T-DXd alone vs. T-DXd followed by taxane, pertuzumab, trastuzumab vs. anthracycline, taxane chemotherapy and pertuzumab, trastuzumab	pCR
	DESTINY-Breast12		2nd or 3rd line with CNS metastasis	3	T-DXd	ORR (cohort 1)
						PFS (cohort 2)
	DEBBRAH (Cohort 1)		≥2-line with CNS metastasis	2	T-DXd	PFS
Trastuzumab Duocarmazine	TULIP		≥3rd line metastatic	3	Trastuzumab duocarmazine vs. physician’s choice of therapy	PFS
**HER2-low, Hormone receptor-positive (HR+)**						
T-DXd	HER2-low (HR+)	TRIO-US B-12/TALENT	Neoadjuvant	2	T-DXd vs. T-DXd plus anastrozole	pCR
	Includes HER2+/HER2-low/HER2− (HR+ and HR−)	DAISY	≥1 chemotherapy in the metastatic setting	2	T-DXd monotherapy	ORR
	HER2-low/HER2− (HR+)	DESTINY Breast-06	2nd or 3rd line metastatic (no prior chemotherapy in metastatic setting)	3	T-DXd vs. physician’s choice of therapy	PFS
Trastuzumab Duocarmazine	HER2-low (HR+)	NCT02277717	Metastatic	1	Trastuzumab duocarmazine	Safety, Pharmacokinetics and Efficacy
**HER2-negative**						
SG	HER2− (HR+/HR−)	SASCIA	Adjuvant setting (residual disease post-neoadjuvant chemotherapy)	3	SG vs. physician’s choice of therapy	IDFS
	HER2− (HR+)	NCT04448886	1st or 2nd line metastatic	2	SG plus pembrolizumab vs. SG alone	PFS
Dato-DXd	HER2− (HR+)	TROPION Breast-01	2nd or 3rd line metastatic	3	Dato-DXd vs. physician’s choice of therapy	PFS
						OS
**Triple Negative (TNBC)**						
SG	NeoSTAR		Neoadjuvant	2	SG vs. SG plus pembrolizumab	pCR
	SASCIA		Adjuvant setting	3	SG vs. physician’s choice of therapy	IDFS
			(residual disease post-neoadjuvant chemotherapy)			
	ASCENT-04		1st line metastatic	3	SG and pembrolizumab vs. physician’s choice of therapy and pembrolizumab	PFS
			(PD-L1-positive)			
	ASCENT-03		1st line metastatic	3	SG vs. physician’s choice of therapy	PFS
			(PD-L1-negative)			
Dato-DXd	TROPION Breast-03		Adjuvant (residual disease post-neoadjuvant chemotherapy)	3	Dato-DXd ± durvalumab vs. physician’s choice of therapy	IDFS
	BEGONIA		1st line metastatic	1–2	Durvalumab plus novel therapies (including Dato-DXd)	Safety, Pharmacokinetics and Efficacy
	TROPION Breast-02		1st line metastatic	3	Dato-DXd vs. physician’s choice of chemotherapy of therapy	PFS
			(PD-L1-negative)			OS
	TROPION Breast-05		1st line metastatic	3	Dato-DXd plus durvalumab	PFS
			(PD-L1-positive)			

Dato-DXd: Datopotamab deruxtecan; HER2-negative: Human epidermal growth factor receptor 2 negative; HER2-positive: Human epidermal growth factor receptor 2 positive; HR+: Hormone Receptor positive; IDFS: Invasive disease-free survival; ORR: Overall response rate; OS: Overall survival; pCR: Pathological complete response; PFS: Progression free survival; SG: Sacituzumab govitecan; TNBC: Triple negative breast cancer. T-DXd: Trastuzumab deruxtecan.
